# Validity of the Common Cold Questionnaire (CCQ) in Asthma Exacerbations

**DOI:** 10.1371/journal.pone.0001802

**Published:** 2008-03-19

**Authors:** Heather Powell, Joanne Smart, Lisa G. Wood, Terry Grissell, Darren R. Shafren, Michael J. Hensley, Peter G. Gibson

**Affiliations:** 1 Department of Respiratory and Sleep Medicine, Hunter Medical Research Institute, John Hunter Hospital, New Lambton, New South Wales, Australia; 2 School of Medical Practice and Population Health, The University of Newcastle, Callaghan, New South Wales, Australia; 3 Picornaviral Research Unit, Royal Newcastle Hospital, Newcastle, New South Wales, Australia; University of Oxford, United Kingdom

## Abstract

**Background:**

The common cold questionnaire (CCQ) is used to discriminate those with and without a viral infection. Its usefulness in people with acute asthma is unknown. Our aim was to assess the ability of the CCQ to detect viral infection and to monitor recovery during a viral induced asthma exacerbation and confirmed by virological testing.

**Methodology/Principal Findings:**

We studied subjects (≥7 yrs) admitted to hospital with acute asthma and diagnosed as positive (n = 63), or negative to viral infection (n = 27) according to molecular and virological testing from respiratory samples. CCQ, asthma history and asthma control questionnaires were completed and repeated 4–6 weeks later. Sensitivity, specificity, and response to change of the CCQ were assessed by receiver operator curve (ROC) analysis and effect size calculation respectively. The CCQ did not discriminate between viral and non-viral infection for subjects with asthma (sensitivity = 76.2%; specificity = 29.6%). ROC analysis could not differentiate between positive or negative virus in subjects with asthma. The CCQ had a large response to change following recovery (effect size = 1.01). 39% of subjects recovering from viral exacerbation remained positive to virological testing at follow-up despite improvement in clinical symptoms. The CCQ reflected clinical improvement in these subjects, thus providing additional information to complement virological testing.

**Conclusions/Significance:**

The CCQ is a useful instrument for monitoring response to viral infection in people with asthma. Reliable differentiation between viral and non-viral asthma exacerbations was not achieved with the CCQ and requires specific virological testing. When combined with virological testing, the CCQ should be a useful outcome measure for evaluating therapies in viral-induced asthma.

## Introduction

Respiratory viral infections are the most common cause of asthma exacerbations in both adults and children [1], [2]. Rhinovirus is the most common virus associated with the common cold and is also the most common virus implicated in asthma exacerbations [1], [2]. There is a need to quantify cold symptoms in the assessment of an asthma exacerbation, both to detect viral causes and to monitor progress. These measures are especially needed to evaluate responses in treatment studies. Several scales have been developed to discriminate those with and without a viral infection [3], monitor symptoms in response to treatment of the common cold [4], and to assess the impact of symptoms on quality of life [5], [6] and disease severity [7], [8] in people without asthma. However, the ability of these questionnaires to discriminate viral infection in acute asthma from a non-infectious exacerbation is not known, and their responsiveness in acute asthma is also unknown. This needs to be directly evaluated since the overlap between symptoms of asthma and common cold symptoms could limit the utility of common cold questionnaires in an asthma exacerbation. Such measures may be particularly important in the assessment of anti-viral and anti-inflammatory therapies in asthma exacerbations. The aims of this study were to assess whether the common cold questionnaire (CCQ) [9] could detect a respiratory viral infection during asthma exacerbation in both adults and children, and to describe the ability of the CCQ to monitor recovery from a viral induced asthma exacerbation.

## Materials and Methods

### Subjects

Eligible subjects were participants in a study of virus-induced asthma where the common cold questionnaire was administered and samples were collected for viral detection by molecular diagnosis. Adults and children ≥7 years admitted to John Hunter Hospital (Newcastle, Australia) experiencing an acute exacerbation of asthma were recruited between February 2001 and May 2005 (n = 90), a subset of which have been previously reported [10]. Exclusion criteria were age less than 7 years.

### Design

Participants attended for two study visits. Firstly during the acute admission for asthma, and secondly at recovery, 4–6 weeks later. At each visit participants completed the common cold questionnaire (CCQ) [3], [9], information on asthma history, an asthma control questionnaire[11], provided nasal/throat swabs, and underwent skin prick allergen testing and spirometry. Induced sputum was collected after ultrasonic nebulisation of isotonic saline as previously described [12], [13], [14]. Subjects were diagnosed as positive or negative to respiratory viral infection according to molecular and virological testing [10]. Written informed consent was obtained from all participants in this study, which was approved by the Hunter Area Health Service and University of Newcastle Research Ethics Committees.

### The Common Cold Questionnaire

The CCQ used in this study was adapted from that used as part of the Common Cold Unit's standard protocol [3], [9]. It records symptoms across four domains: general symptoms; nasal symptoms; throat symptoms; and chest symptoms (Table 1). Symptoms are scored using a 4-point category scale as none = 0, mild = 1, moderate = 2 or severe = 3 and summed with a maximum total score of 27. Subjects were classified by CCQ as ‘no virus’ if there were no symptoms, as a ‘possible virus’ if they had mild symptoms in one domain plus a cough, and as a ‘probable’ virus if there were moderate symptoms in at least two domains or mild symptoms in three domains. The asthma history questionnaire detailed the age of diagnosis and the history of the subject's asthma over the past year. This included the number of exacerbations, doctor and hospital visits, episodes of worsened asthma and the number of days missed from work or school due to asthma. Asthma control was assessed over the previous week using an asthma control questionnaire that scored symptoms, activity limitation and rescue beta agonist use on a 0–6 scale[11].

**Table 1:** Common Cold Questionnaire

In the 2 days prior to admission with asthma, has the subject experienced any of the following symptoms? Please circle the severity of the symptoms experienced.					
**General Symptoms:**	1. Fevers	None	Mild	Moderate	Severe
	2. Chills	None	Mild	Moderate	Severe
	3. Muscle pains	None	Mild	Moderate	Severe
**Nasal Symptoms:**	1. Watery eyes	None	Mild	Moderate	Severe
	2. Runny nose	None	Mild	Moderate	Severe
	3. Sneezing	None	Mild	Moderate	Severe
**Throat Symptoms:**	1. Sore throat	None	Mild	Moderate	Severe
**Chest Symptoms:**	1. Cough	None	Mild	Moderate	Severe
	2. Chest pain	None	Mild	Moderate	Severe
A ‘probable’ viral infection is where there are moderate symptoms noted in at least 2 of the above 4 categories or mild symptoms noted in 3 or more categories.					
A ‘possible’ viral infection is where mild symptoms are noted in one category plus a cough. Scoring: none = 0, mild = 1, moderate = 2, severe = 3. Total score equals the sum of all scores					

### Specimen Processing

Lower respiratory portions were selected from induced sputum samples and placed in an RNA lysis buffer (Buffer RLT-Qiagen, Hilden, Germany) as previously described [10]. Nasal swabs and throat swabs were also immersed in Buffer RLT. Extraction and purification of sputum, swab RNA was performed using the RNeasy kit (Qiagen) as per manufacturer's instructions. RNA was then reverse-transcribed to total cDNA using random primers and the Superscript II RT kit (Invitrogen, Carlsbad, USA).

Samples were assayed for the presence of rhinovirus (RV), enterovirus (EV), influenza virus types A and B (IFA, IFB), respiratory syncytial virus types A and B (RSVA, RSVB), non-SARS coronavirus (CoV) and human metapneumovirus (MPV) virus RNA transcripts. Using the gel-based PCR assays [10] or real-time ‘TaqMan’ methodology PCR assays (RV[15], EV[16], RSVA & RSVB[17], hMPV[18], CoV). All TaqMan assays proceeded using 12.5% of the cDNA product and the Eppendorf RealMasterMix Probe ROX kit (Eppendorf AG, Hamburg, Germany), all with the same cycling parameters, namely: 2 min at 95°C to activate HotMaster Taq DNA polymerase and 40 cycles of 95°C for 15 sec followed by 1 min at 60°C (ABI 7500 cycler; Applied Biosystems, Foster City, CA, USA). Subjects were considered virus positive if a virus was detected by direct molecular detection in sputum, swab (nasal or throat) or saliva.

### Statistical Analysis

Analysis was performed using Stata 7 (Stata Corporation, College Station, Texas USA), with results presented as median (interquartile range) or n (%) as appropriate. Differences in subject characteristics and common cold score between virus positive and virus negative groups were determined (significance = p<0.05) from non-parametric Wilcoxon ranksum test, Kruskall Wallis test or Fisher's exact test and Chi-square test. Paired analyses were conducted using Wilcoxon sign rank test.

Receiver operator curve (ROC) analyses were used to evaluate different diagnostic cut-off levels for the CCQ score for all subjects and for adults (≥16 years) and children (<16 years) separately. Sensitivity, specificity, positive and negative predictive values and likelihood ratios were used to describe the discriminant validity of the CCQ for determining a ‘probable virus’ versus a ‘no virus’ or ‘possible virus’.

The ability of the CCQ to respond to a change in health status in virus infected subjects was evaluated by calculating the effect size as described by Cohen et al [19]. The effect size is the mean difference between the scores at the first and follow up visits divided by the standard deviation at the first visit. An effect size of 0.8 or more is considered a large response to change [19].

## Results

Data were collected 1.3 (0.8), mean (SD), days post admission. Respiratory virus infection was present in 63 (70%) subjects, with a negative respiratory virus result in 27 (30%) subjects. The viruses detected were: rhinovirus (n = 52, 83%), enterovirus (n = 18, 29%), RSV (n = 1, 2%), Influenza A (n = 5, 8%) and Influenza B (n = 2, 3%). In 14 subjects dual viruses were detected and in one subject three different viruses were detected. Table 2 describes the subject characteristics for the virus positive and virus negative groups. There was no difference in the proportion of subjects with atopy, smoking status or gender between the groups. Those with a positive virus result had a lower percent predicted FEV_1_, were younger than the virus negative group and had a similar asthma control score (Table 2).

**Table 2:** Characteristics of subjects with Asthma Exacerbations

	*Virus negative by PCR*	*Virus positive by PCR*	*P-value* *
n	27	63	
Age, median (IQR)	28.7 (16.5, 52.6)	13.6 (9.8, 37.1)	0.007
Sex (M/F)	11/16	22/41	0.600
Smoker, n (%)	5 (19.2)	8 (13.3)	0.483
Atopy, n (%)	15 (88.2)	44 (89.8)	1.0
FEV_1_ percent predicted, median (IQR)	70.0 (56.0, 80.9)	62.0 (46.4, 70.0)	0.075
Asthma control score, mean (SD)	3.26 (1.34)	2.84 (1.34)	0.183 #

IQR: interquartile range

* chi-square test or Wilcoxon ranksum test; ^#^ Student's t test

### Common Cold Questionnaire Scores

The CCQ scores by domain and subject group are described in Table 3. The questionnaire identified a similar proportion in both the viral and non-viral groups as having a ‘probable virus’ or ‘no virus’ (p = 0.802). Total score, general, nasal, throat and chest domain scores were similar for both virus positive and virus negative groups (Table 3). However a significantly higher total score was evident in the virus positive group for adults but not children.

**Table 3:** Common Cold Questionnaire (CCQ) Scores During Asthma Exacerbation for all Subjects.

		*Virus negative by PCR*	*Virus positive by PCR*	*P-value*
n		27	63	
CCQ	No virus, n(%)	4 (14.8)	8 (12.7)	0.802*
	Possible virus, n(%)	4 (14.8)	7 (11.1)	
	Probable virus, n(%)	19 (70.4)	48 (76.2)	
Total score	All subjects	7 (5, 13)	9 (5, 13)	0.628
	Adults (n = 48)	6 (1, 10)	10 (7, 13)	0.009
	Children (n = 42)	4 (2, 11)	7.5 (4, 11)	0.449
General symptoms score		1 (0, 5)	1 (0, 3)	0.711
Nasal Symptoms score		2 (0, 4)	3 (1, 5)	0.219
Throat symptoms score		1 (0, 2)	1 (0, 2)	0.156
Chest symptoms score		2 (2, 4)	3 (1, 5)	0.921

Results are median (IQR), Wilcoxon ranksum test. * Fisher's exact test

**Figure 1:**
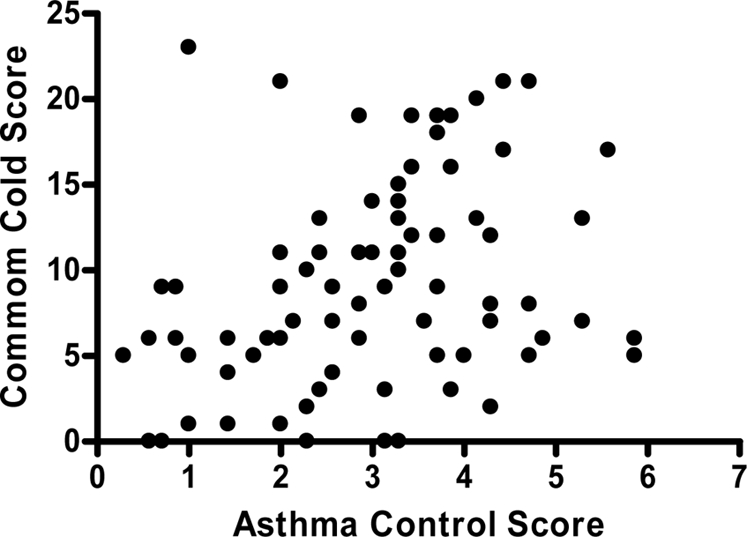
Correlation of Common Cold Total Score and Asthma Control Score for all Subjects

The total CCQ score demonstrated a weak and clinically significant correlation to asthma control score (spearman's rho (95%CI) = 0.35 (0.14 to 0.53), Figure 1). There was no correlation between total CCQ score and percent-predicted FEV_1_ (spearman's rho = 0.03, p = 0.821) and no significant difference in score between exacerbations classified as mild, moderate, or severe based on their percent-predicted FEV_1_ at admission (≥80%; ≥60<80%; <60%) (p = 0.08).

### CCQ and Viral Detection

The ability of the CCQ to detect a respiratory virus infection in subjects with asthma, adults and children is displayed in Table 4. The CCQ was scored and results categorised as a ‘probable virus infection’ compared to a ‘no infection’ or ‘possible virus infection’ result. For all subjects, both adults and children, moderate sensitivity was traded against poor specificity and low negative predictive values (Table 4). A CCQ result of ‘probable virus infection’ did have a good positive predictive value for viral infection in children.

**Table 4:** Performance Characteristics of the Common Cold Questionnaire for Virus Infection+

Tests	Sensitivity (95%CI)	Specificity (95%CI)	PPV (95%CI)	NPV (95%CI)	+ve LR (95%CI)	-ve LR (95%CI)
All asthma	76.2% (63.5 to 85.6)	29.6% (14.5 to 50.3)	71.6% (59.1 to 81.7)	34.8% (17.2 to 57.2)	1.08 (0.82 to 1.43)	0.80 (0.43 to 1.49)
Adults	85.2 % (65.4 to 95.1)	23.8% (9.1 to 47.5)	59.1% (42.2 to 74.0)	55.6% (22.7 to 84.7)	1.12 (0.84 to 1.49)	0.62 (0.19 to 2.07)
Children	69.4% (51.7 to 83.1)	50.0% (13.9 to 86.1)	89.2% (70.6 to 97.2)	21.4% (5.7 to 51.2)	1.39 (0.61 to 3.18)	0.61 (0.27 to 1.39)

PPV: positive predictive value

NPV: negative predictive value

LR: Likelihood ratio

+ Common cold questionnaires were scored as ‘probable virus’ or ‘no or possible virus’ as per methods.

**Figure 2:**
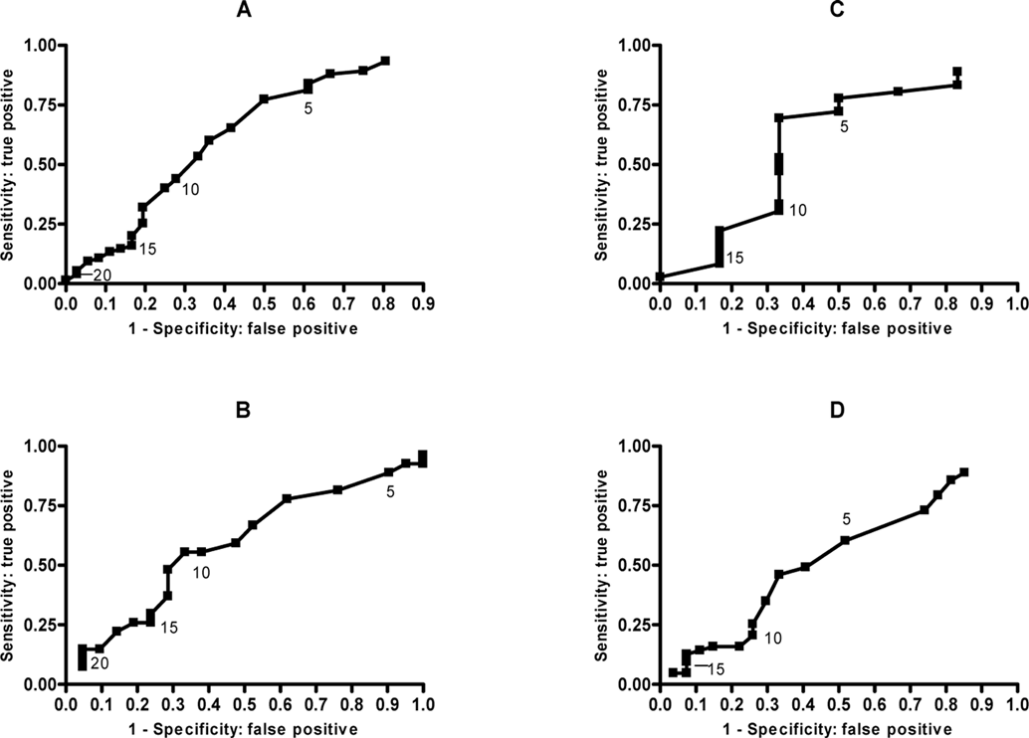
Receiver Operator Curves for Common Cold Score and Virus Result. A: CCQ (all asthma); B: CCQ (adults); C: CCQ (children); D: CCQ (excl chest symptoms).

ROC analysis was unable to determine an appropriate CCQ score to discriminate between a virus positive and virus negative result for all subjects or adults or children separately (Figure 2a, 2b, 2c).

A sensitivity analysis was conducted by excluding the chest symptom domain from the ROC analysis to account for the possible confounding effect that these symptoms may have in people with asthma with or without a virus (Figure 2d). This did not improve the ability of the CCQ to discriminate between virus positive and virus negative subjects (AUC = 0.533, p = 0.619).

### Response to Change in Health Status

**Figure 3:**
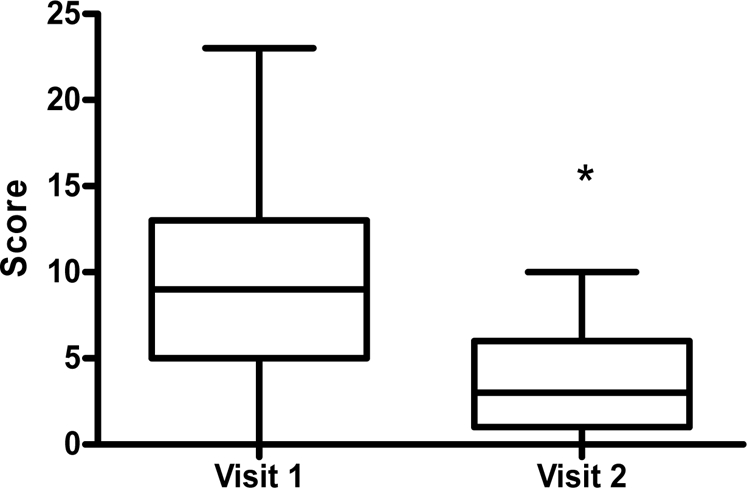
Common Cold Questionnaire Median (IQR) Total Score for Visits 1 & 2 for Subjects with Asthma Exacerbation and Viral Respiratory Infection. * P = 0.0001

The CCQs response to change following recovery from a respiratory virus infection was large (Figure 3). The effect size calculated between the first and follow up visit at 4–6 weeks for 33 subjects who were virus positive was 1.01. This was in conjunction with significant clinical improvements in FEV_1_ percent predicted (mean (SD) 21.4(19.1)%, p<0.0001) and asthma control score (−1.6 (1.2), p<0.0001). However on molecular testing 13 (39%) remained positive to virus. These 13 subjects who remained virus positive at visit 2, also had improved clinical outcomes including CCQ total score, FEV_1_ percent predicted and asthma control score (Table 5).

**Table 5:** Change in clinical markers during acute exacerbation and at 4–6 week follow up for subjects PCR positive at visit 1 and 2 (n = 13). Results are median (IQR), Wilcoxon sign rank test.

	*Visit 1*	*Visit 2*	*P-value*
FEV_1_ percent predicted	65.0 (15.0)	88.5 (14.1)	0.002
CCQ total score	9 (6, 13)	3 (1, 5)	0.023
Asthma control score	2.3 (1.7)	1.0 (0.8)	0.019

## Discussion

In this study we have been the first to examine the validity of the CCQ to differentiate between viral and non-viral exacerbations of asthma in both adults and children using molecular diagnostics to confirm the viral infection. Although median CCQ scores were higher in viral infection in adults, the CCQ did not discriminate sufficiently well between viral and non-viral exacerbations of asthma. In contrast the CCQ performed very well in monitoring recovery of the viral infection, providing additional information to an asthma symptom score. This suggests a useful role for the CCQ in treatment studies, when combined with virological diagnosis.

The majority of our subjects were recruited as inpatients with an acute exacerbation of asthma and were a population with significant disease, in whom discrimination of viral and non-viral causes would be useful. The CCQ performed poorly in discriminating between a viral and non-viral exacerbation in people with acute asthma. The poor discrimination of the CCQ in acute asthma was due to the low specificity of the CCQ in this setting. Many subjects scored as ‘probable virus’ according to the CCQ but were negative to virus by molecular diagnostic testing. The poor fitting ROC curve indicated that there was no obvious CCQ score that was diagnostic (Figure 2a).

This is perhaps not surprising, given the limitations to viral diagnostics, even when using PCR. It is therefore possible that respiratory viruses could have been responsible for the exacerbation, but went undetected either because of assay sensitivity or infection with as yet unidentified viruses. The lack of discrimination in CCQ score was evident for both adults and children. To exclude the possibility that the common symptoms of cough in asthma may be confused for cold symptoms, further ROC analysis was conducted excluding the chest symptoms domain. However this did not improve the diagnostic ability of the CCQ in asthma exacerbations (Figure 2d).

The low specificity for the CCQ in asthma may be due to several additional factors. People with asthma respond to other triggers that can cause nose symptoms such as allergens, and rhinitis is a common comorbidity in asthma. Chest symptoms are also a feature of an asthma exacerbation, and constitute one of the domains of the CCQ. Thus non-infectious exacerbations of asthma could cause chest symptoms and reduce the specificity of the CCQ. Recent data indicate an altered pathogenesis of viral infection in asthma compared to controls. RV infection is considered to be an upper airway infection. However, people with asthma experience lower respiratory symptoms more often in viral infection [20] and RV is frequently isolated from the lower respiratory tract in asthma [21], [22], [23]. This altered pathogenesis may be due to impaired innate antiviral responses in the respiratory epithelium in asthma [24]. The result is a greater overlap between common cold and asthma symptoms. Consequently there are several possible explanations for the limited discriminant validity of the CCQ in asthma.

The relationship between CCQ scores and asthma severity was assessed using two severity measures: percent-predicted FEV_1_ and asthma control score. There was no relationship between CCQ score and severity of airflow obstruction (FEV_1_) during the exacerbation; however, CCQ was weakly correlated to asthma control score. This may be due to an association between asthma symptoms, severity and CCQ in acute asthma, or an overlap in symptoms assessed by the two questionnaires.

The CCQ did prove useful in monitoring symptoms over time in asthma. The response of the CCQ to a change in health status exceeded the large response (>0.8) considered to be clinically useful by Cohen [19]. The effect size indicates that in people with asthma the CCQ has a high response to change following a viral infection. This validates the use of the CCQ to monitor recovery and indicates the potential usefulness of this tool in monitoring clinical recovery post viral infection, and as an evaluative tool in treatment studies [25].

At recovery, 39% of subjects remained positive to virological testing despite an improvement in clinical symptoms. The CCQ may be a more sensitive reflection of clinical improvement following viral infection in people with asthma and may provide additional information when monitoring response to treatment.

The CCQ is an attractive instrument for monitoring response to viral infection in asthma due to viral infections. However the CCQ is not able to reliably differentiate between viral and non-viral asthma exacerbations. The combination of viral diagnosis and the CCQ should prove useful for the evaluation of antiviral and other therapies in asthma exacerbations.
